# Human adenovirus (HAdV) infection in children with acute respiratory tract infections in Guangzhou, China, 2010–2021: a molecular epidemiology study

**DOI:** 10.1007/s12519-022-00590-w

**Published:** 2022-07-21

**Authors:** Yi Chen, Tao Lin, Chang-Bing Wang, Wan-Li Liang, Guang-Wan Lian, Mark Zanin, Sook-San Wong, Xin-Gui Tian, Jia-Yu Zhong, Ying-Ying Zhang, Jia-Hui Xie, Ling-Ling Zheng, Fei-Yan Chen, Run Dang, Ming-Qi Zhao, Yi-Yu Yang, Rong Zhou, Bing Zhu

**Affiliations:** 1 Center Laboratory, Guangzhou Women and Children’s Medical Center, Guangzhou Medical University, Guangzhou, 510120 China; 2 State Key Laboratory of Respiratory Diseases, Guangzhou Institute of Respiratory Health, Guangzhou Medical University, Guangzhou, China; 3 Intensive Care Unit, Guangzhou Women and Children’s Medical Center, Guangzhou Medical University, Guangzhou, 510120 China; 4 Guangzhou Medical University, Guangzhou, 510120 China

**Keywords:** Acute respiratory tract infection, Children, Human adenovirus, Severe acute hepatitis, Southern China

## Abstract

**Background:**

Human adenovirus (HAdV) infection can cause a variety of diseases. It is a major pathogen of pediatric acute respiratory tract infections (ARIs) and can be life-threatening in younger children. We described the epidemiology and subtypes shifting of HAdV among children with ARI in Guangzhou, China.

**Methods:**

We conducted a retrospective study of 161,079 children diagnosed with acute respiratory illness at the Guangzhou Women and Children’s Medical Center between 2010 and 2021. HAdV specimens were detected by real-time PCR and the *hexon* gene was used for phylogenetic analysis.

**Results:**

Before the COVID-19 outbreak in Guangzhou, the annual frequency of adenovirus infection detected during this period ranged from 3.92% to 13.58%, with an epidemic peak every four to five years. HAdV demonstrated a clear seasonal distribution, with the lowest positivity in March and peaking during summer (July or August) every year. A significant increase in HAdV cases was recorded for 2018 and 2019, which coincided with a shift in the dominant HAdV subtype from HAdV-3 to HAdV-7. The latter was associated with a more severe disease compared to HAdV-3. The average mortality proportion for children infected with HAdV from 2016 to 2019 was 0.38% but increased to 20% in severe cases. After COVID-19 emerged, HAdV cases dropped to 2.68%, suggesting that non-pharmaceutical interventions probably reduced the transmission of HAdV in the community.

**Conclusion:**

Our study provides the foundation for the understanding of the epidemiology of HAdV and its associated risks in children in Southern China.

## Introduction

Human adenoviruses (HAdVs) cause a wide range of illnesses in individuals of all ages, including acute respiratory infections (ARIs), gastroenteritis, conjunctivitis, cystitis, and meningoencephalitis. Although HAdV infections often range from mild to moderate in severity, cases of severe pneumonia and death in otherwise healthy adults also have been reported [1, 2]. Life-threatening respiratory disease related to HAdV infections mostly occurs in younger children, the elderly, and individuals with severely compromised immune systems [3–5]. For example, HAdV-associated ARI is one of the most common causes of morbidity and mortality in children. HAdV-3, -4, -14, -55 and -7 are the major HAdV subtypes associated with ARI in children and in adults globally. The major HAdV subtypes detected in various countries and regions differ and change with time [6–9]. In China, HAdV-3 and -7 are the major subtypes, and they alternate in predominance from year to year. The subtype distributions in Southern and Northern China were occasionally not synchronous [10–13].

In the second half of 2018, we found that the number of pediatric patients with ARI coming to pediatric hospitals was increasing, as well as the number of severe cases. This situation became more prominent in 2019 when the number of infections and severe cases had reached a record high. The purpose of this retrospective study was to determine the prevalence, epidemiology and subtypes of HAdV circulating among children with ARI in Guangzhou, Southern China during the period 2010–2021. All the specimens were collected in Guangzhou Women and Children’s Medical Center (GWCMC), a large pediatric and women’s hospital that has a regional children’s medical service network in Southern China. The hospital has about 2000 beds distributed in seven branches, receives over 4,700,000 pediatric outpatient person-times and admits 140,000 inpatients each year from Guangzhou as well as from other cities in Southern China. Therefore, our data provide a reasonable approximation of the HAdV activity in Southern China.

## Methods

### Patient cohort

Data were collected retrospectively from 161,079 children ≤ 14 years old who were diagnosed with ARI in the outpatient department, general ward and intensive care units (ICU) of the GWCMC between 1 January 2010 and 31 December 2021. Cases with repeated detections were not counted. Throat swabs or sputum collected from children were tested for HAdV, and samples from patients in ICU were detected for multiple respiratory viruses. We excluded the ICU cases who tested positive for influenza virus, respiratory syncytial virus, enterovirus, and known or suspected to have active tuberculosis, severe concomitant disease (chronic pulmonary disease except for asthma, severe cardiovascular disease, neoplasia, and kidney or liver disease), primary immunodeficiency, acquired immunodeficiency syndrome and patients taking immunosuppressive medications. Severe HAdV pneumonia was diagnosed when one of the following conditions was present: hypoxemia, requiring either invasive mechanical ventilation or noninvasive positive pressure ventilation and fluid refractory shock.

Retrospective analysis of demographic data of HAdV infection was from 2014 to 2019, and the monthly incidence of HAdV infections was between 2012 and 2021.

### Ethical approval

This project was approved by the Ethics Committee of the GWCMC and was carried out in accordance with the principles of the Declaration of Helsinki. Clinical data and clinical specimens were de-identified and anonymous.

### Clinical specimens

Throat swabs were collected in 2.50 mL of viral transport medium whereas sputum was collected by aspiration. All specimens were delivered to the central diagnostic laboratory of GWCMC and were processed within 24 hours of collection.

### Detection of adenovirus and molecular typing with PCR

HAdV molecular typing was performed on 557 HAdV-positive children randomly selected in respiratory wards from 2012 to 2019 and on 121 children diagnosed with severe adenovirus pneumonia in the ICU from 2014 to 2019. Total nucleic acid was extracted by the automated nucleic acid purification system Expure-20 (Shenzhen Huiyan Kechuang Biotechnology Co., China) using a total nucleic acid isolation kit (Shenzhen Huiyan Kechuang Biotechnology Co., China) according to the manufacturer's instructions. HAdV nucleic acid was detected by real-time quantitative polymerase chain reaction (RT-qPCR). Molecular typing of HAdV-3, -4, -7, -11, -14 and -55 was performed using the Taqman real-time PCR kit according to the manufacturer’s protocol (Guangzhou HuYanSuo Medical Technology Co., China). Furthermore, the hypervariable regions (HVRs) of the HAdV *hexon* gene were amplified by PCR using the HVR forward (HVRF) and HVR reverse (HVRR) primers to amplify the 1.6 kbp fragment of the seven HVRs, as described previously [14]. The amplicon was submitted for sequencing (Invitrogen, Guangzhou). The positive samples were classified as HAdV-3, HAdV-7, or other HAdV subtypes.

### HAdV genome sequencing

The *hexon* of HAdV was amplified and then the obtained sequences were used for the Basic Local Alignment Search Tool (BLAST) search (NCBI). The whole genome of HAdV was sequenced using Sanger sequencing. Assembly of the complete sequences of the HAdV genomes was accomplished using SeqMan software from the Lasergene package, as described previously [3]. Multiple sequence alignments and phylogenetic tree construction were performed using Molecular Evolutionary Genetics Analysis (MEGA) version 11. The multiple sequence alignments were then revised using Clustal W. Phylogenetic trees were constructed by the Unweighted Pair Group Method with Arithmetic (UPGMA) method with 1000 bootstrap replicates, and default settings were used for all other parameters.

### Detection of other common respiratory pathogens with RT-qPCR among ICU specimens

The children admitted to ICU were tested for HAdV and 10 other respiratory pathogens: influenza A virus (infA), influenza B virus (infB), parainfluenza virus (PIV), respiratory syncytial virus (RSV), enterovirus (EV), human metapneumovirus (hMPV), bocavirus (BOV), rhinovirus (RHV), *Mycoplasma pneumoniae* (*MP*), and *Chlamydia pneumonia* (*CP*) using a commercially available Taqman RT-qPCR kit (Guangzhou HuYanSuo Medical Technology Co. LTD; China).

### Statistical analysis

Chi-square tests followed by post-hoc comparisons using Bonferroni’s adjustment were used to assess statistical significance. All tests were two-tailed with *P* < 0.05 considered to be statistically significant.

## Results

### Number of ARI cases associated with HAdV infection in children increased in 2018 and 2019

**Fig. 1:**
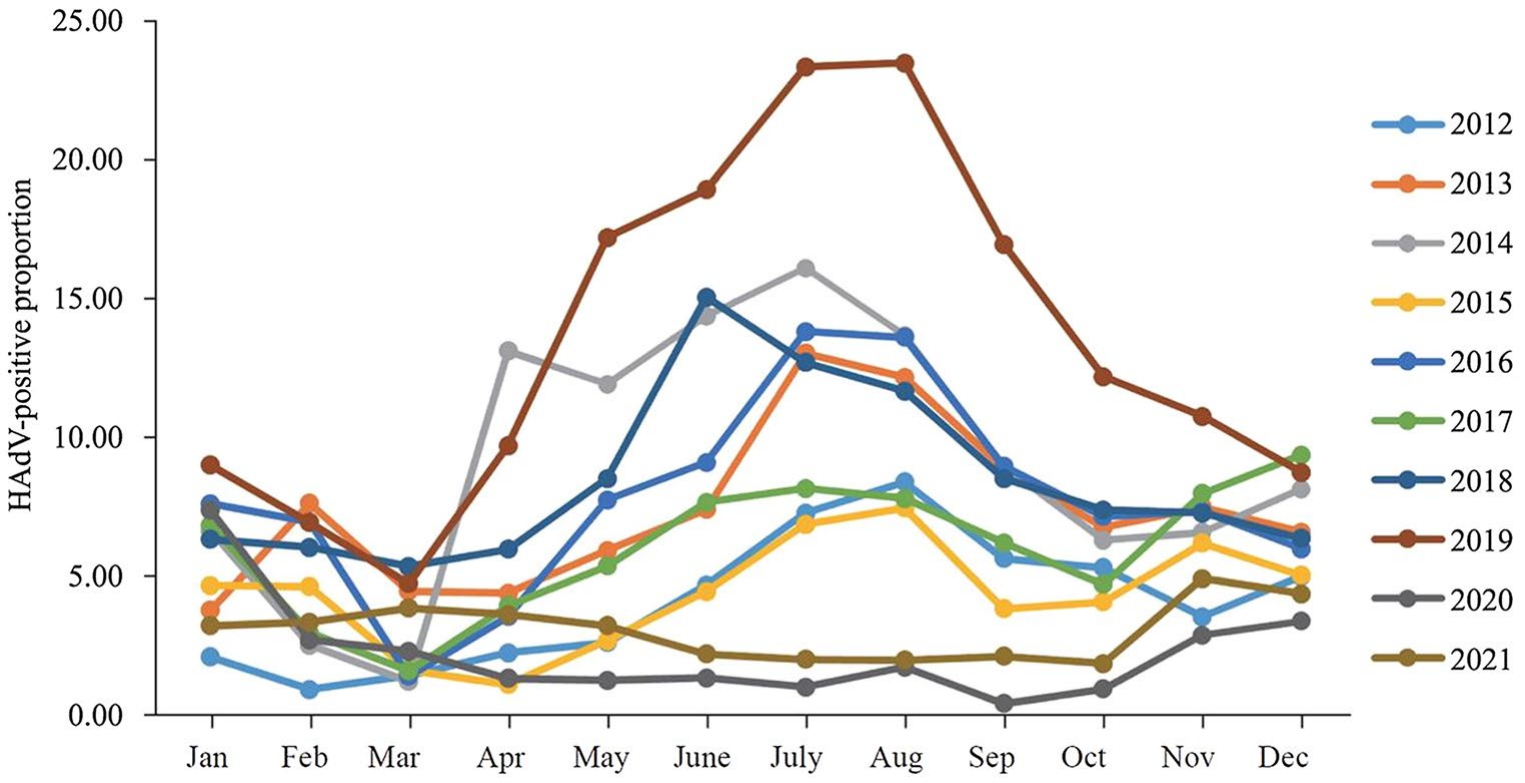
Monthly incidence of human adenovirus (HAdV)-positive cases among children with acute respiratory tract infection (ARI) at GWCMC in Guangzhou between January 2012 and December 2021. *GWCMC* Guangzhou women and children’s medical center

Based on our retrospective data collected from pediatric patients diagnosed with ARI at GWCMC, the number of laboratory-confirmed HAdV cases increased from 4543 in 2010 to 39,938 in 2019. Before the COVID-19 pandemic, the lowest annual frequency was in 2012 (3.92%) and the highest annual frequency in 2019 (13.58%) (Fig. 1). Among patients who were tested positive for HAdV between 2010 and 2018, the hospitalization frequency was 71.48% to 92.35%. Prior to 2019, when the frequency of positivity was highest, the proportion of HAdV-positive inpatients fell to 62.14% and the proportion of outpatients rose to 37.86%. This was attributed to the increased number of infections in the general population and to the hospital reaching full in-patient capacity during this time.

Within the 2010–2021 time period, demographic data were available only for 102,469 patients from 2014 to 2019 (Table 1). The total HAdV positivity was 9.70%, with no significant differences detected by gender. The frequency of HAdV-positivity in those aged 0–1, 1–3, 3–6 and 6–14 years were 8.99%, 11.18%, 7.55% and 12.52%, respectively, with the positivity in 1–3- and 6–14-year-olds being significantly greater compared to the other age groups (*P < *0.001).

**Table 1:** Positivity rate of human adenovirus (HAdV) infections in children seeking treatment at GWCMC for acute respiratory tract infection (ARI) between 2014 and 2019

Variables	Samples tested (%)	HAdV positive (%)	*P* value*
Total	102,469 (100.00)	9965 (9.72)	
Age (y)			< 0.001
0–1	25,822 (25.20)	2321 (8.99^†^)	
1–3	36,171 (35.30)	4046 (11.18^‡^)	
3–6	29,818 (29.10)	2252 (7.55^§^)	
6–14	10,656 (10.40)	1335 (12.52^||^)	
Gender			0.788
Male	66,604 (65.00)	6465 (9.71)	
Female	35,865 (35.00)	3500 (9.76)	

*GWCMC* Guangzhou women and children’s medical center. ^*^Chi-square analysis, followed by Bonferroni corrections was used to calculate the *P* values. The letter ^†^, ^‡^, ^§^, ^∥^ indicated that there was a statistical difference, on the positivity rate amongst the ages

### Monthly incidence of HAdV infections between 2012 and 2021

Based on data collected from January 2012 to December 2021, we studied the monthly incidence of HAdV and found that HAdV was detectable throughout the year (Fig. 1). Before the COVID -19 pandemic, the highest HAdV positivity tended to occur during the summer months between June and September, whereas the lowest positivity occurred between February and March. The lowest HAdV positivity was detected in February 2012 (0.92%) and April 2015 (1.10%), while high positivity was found in July (23.48%) and August (23.62%) of 2019, respectively. In most years HAdV activity peaked between July and August, except in 2018 when it peaked in June. After the COVID-19 pandemic, HAdV activity showed less obvious seasonality, and the positivity frequency was below 5.00% each month between 2020 and 2021.

### Shift in the dominant HAdV subtype from HAdV-3 to HAdV-7

**Fig. 2:**
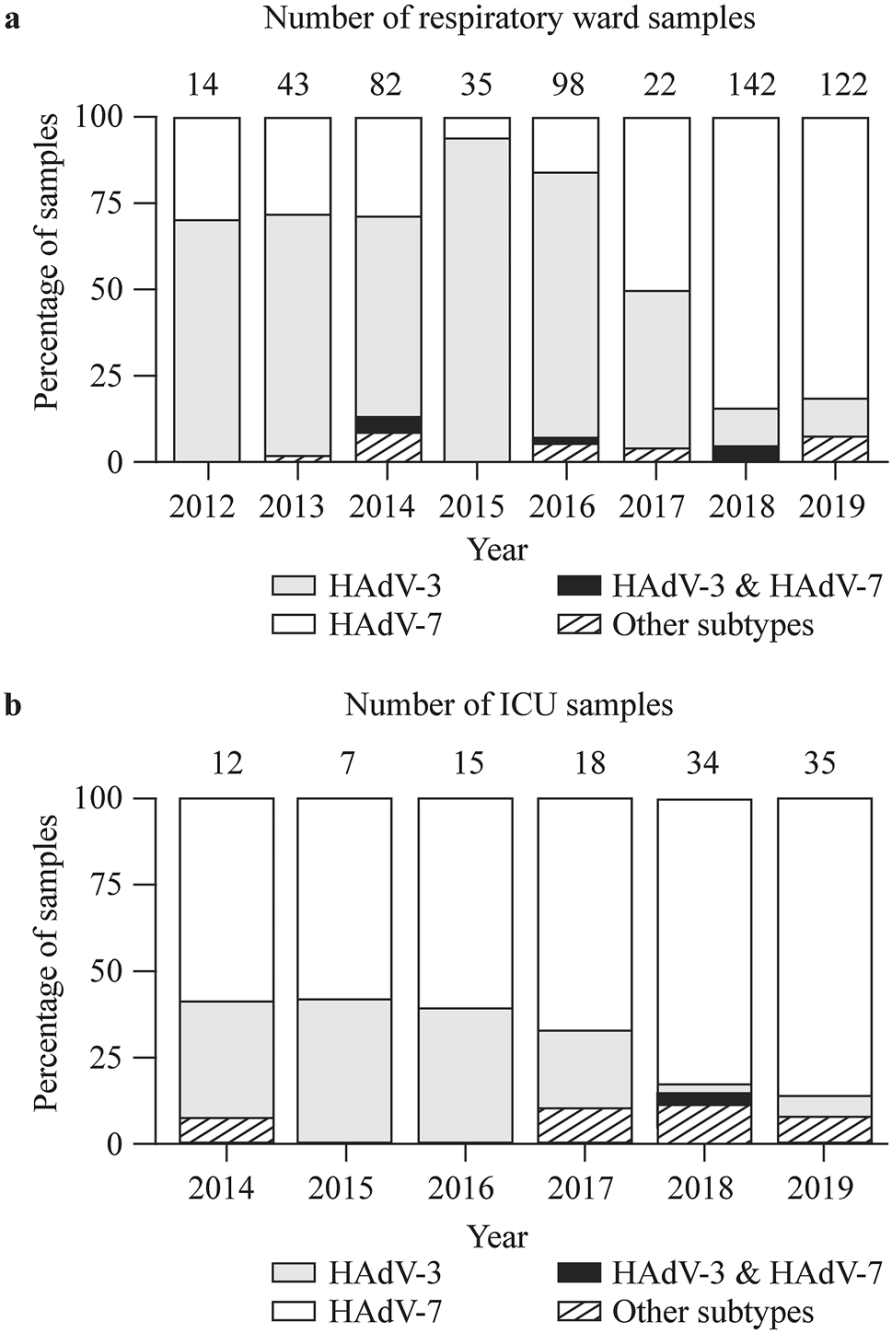
Subtypes of the human adenovirus (HAdV) detected in the respiratory samples in **a** respiratory ward and **b** in those admitted to the intensive care unit (ICU) of GWCMC in the specified years. *GWCMC* Guangzhou Women and Children’s Medical Center

Molecular typing showed that among 557 patients in respiratory wards, the proportion was (1) HAdV-3: 42.01% (234/557), (2) HAdV-7: 51.17% (285/557), (3) other HAdV subtypes: 4.85% (27/557), and HAdV-3 and HAdV-7 co-infection 1.97% (11/557). Among 121 ICU patients, the proportion was (1) HAdV-3: 16.53% (20/121), (2) HAdV-7: 74.38% (90/121), (3) other HAdV subtypes: 8.26% (10/121), and there was one case of HAdV-3 and HAdV-7 co-infection. Overall, the majority of HAdV cases at GWCMC were caused by HAdV-3 and HAdV-7 (Fig. 2a), with relatively rare cases of HAdV-3 and HAdV-7 co-infection being detected in 2014, 2016 and 2018, accounting for 3.66%, 1.03% and 4.93%, respectively. Cases of ARI caused by other HAdV subtypes were also relatively rare (< 10% each year).

From 2012 to 2016, HAdV-3 was the most commonly detected subtype and was responsible for an annual average of 74.30% ± 0.06% of cases (Fig. 2a). HAdV-3 frequency ranged from 58.54% (2014) to 94.29% (2015), while HAdV-7 made up the majority of the remaining cases. However, from 2017 onwards, we observed a change in the dominant subtype detected. The proportion of HAdV-7 and HAdV-3 cases was similar in 2017, at 50.00% and 45.45%, respectively, but in 2018 and 2019, HAdV-7 became the predominant subtype, causing an annual average of 82.45% ± 0.01% of cases (Fig. 2b). Our data from the ICU were collected between 2014 and 2019, and despite a lower overall rate of detection, the HAdV-7 positivity frequency amongst ICU admissions was significantly greater than that of HAdV-3 (60.5% ± 0.02% and 34.6% ± 0.04%, respectively, Fig. 2b). After 2017, HAdV-7 continued to cause a greater proportion of ICU cases compared to other subtypes. Therefore, regardless of the current epidemic strain, HAdV-7 was always dominant in the ICU. Of note, we also detected two cases of HAdV-55 during our study period and a single case of HAdV-14 in 2019 in the ICU.

### Mortality proportion in pediatric cases of ARI was greater in 2018 compared to previous years

With the increasing clinical need for pathogens detection and with awareness of the population seeking more healthcare, especially the 2018/2019 adenovirus epidemic in children in Guangzhou, there was an interannual increase in the number of ARI cases at GWCMC from 4543 in 2010 to 19,338 in 2018 and 39,938 in 2019 (Table 2). The number of ARI cases that tested HAdV positive was also greater in 2018 and 2019 compared to previous years, at 1718 and 5424, respectively, compared to an average of 551 ± 92.70 for previous years. The proportion of ARI cases that tested positive for HAdV was also greater in 2019, at 13.58%, compared to previous years, at 6.37% ± 0.64%. The numbers of HAdV cases in the ICU of GWCMC also increased during 2016, 2017, 2018 and 2019, with 15, 20, 34 and 35 cases, respectively, as did the number of ICU cases that were fatal, with 3, 3, 8 and 15 cases, respectively (Table 2). Of the 29 deaths in the ICU, 27 were HAdV-7 positive and the remaining two were HAdV-3 and HAdV-55. All of these deaths had typical symptoms of adenovirus pneumonia, such as bronchiolitis obliterans (BO) and bronchiectasis. The main cause of death was a respiratory failure (*n *= 24), while the other five cases died due to multiple organ dysfunction syndromes (MODS) and shock. No co-infection with other common respiratory pathogens was detected (detailed in the patient cohort).

**Table 2:** Numbers of pediatric cases of acute respiratory tract infection (ARI) and numbers positive for HAdV that were outpatients, hospitalized and admitted to the intensive care unit (ICU) of GWCMC between 2010 and 2021

Year	ARI cases*	HAdV positive (%)	Outpatient (%)	Hospitalized (%)	ICU cases	Fatal ICU cases (%)
2010	4543	266 (5.86)	28.52	71.48	NA	NA
2011	7312	439 (6.00)	10.31	89.69	NA	NA
2012	5682	223 (3.92)	8.86	91.14	NA	NA
2013	6912	518 (7.49)	7.65	92.35	NA	NA
2014	9668	918 (9.50)	12.81	87.19	NA	NA
2015	10,149	434 (4.28)	13.99	86.01	NA	NA
2016	10,180	773 (7.59)	17.59	82.41	15	3 (0.39)
2017	13,196	837 (6.34)	16.11	83.89	18	3 (0.36)
2018	19,338	1718 (8.88)	20.93	79.07	34	8 (0.47)
2019	39,938	5424 (13.58)	37.86	62.14	35	15 (0.28)
2020	15,804	423 (2.68)	17.86	82.14	4	0
2021	18,357	565 (3.08)	9.94	90.06	10	0

*HAdV* Human adenovirus, *GWCMC* Guangzhou Women and Children’s Medical Center, *NA* not available. ^*^Total number of cases from 2010 to 2021 was 161,079

### Genomic characterization of HAdV from fatal cases

During the course of our study, we noted that there were three particularly severe cases of ARI associated with HAdV infection in the ICU in 2018. To gain a greater insight into the causative viruses, we performed whole viral genome sequencing on samples obtained from these patients. One case was identified as HAdV-55 and the other two were identified as HAdV-7d. The complete genome sequences of these strains were deposited in the GenBank database under accession numbers MK123978.1, MN164629.1 and MN135993.1. In our study, the two HAdV-7d isolates MN164629.1 and MN135993.1 had identical genomes (99.9%) with an earlier strain from a fatal acute respiratory distress case during an outbreak in China in 2009 (HAdV-7 0901 HZ) [15]. These data suggest relatively low mutation rates in the HAdV-7 genome.

### HAdV cases were reduced significantly between 2020 and 2021

In February 2020, when the most stringent control measures against COVID-19 were implemented in Guangzhou, HAdV positivity was only 2.68% (out of 15,804 total samples tested) and was the lowest since 2010. The morbidity dropped to a very low level and without fatal cases (Table 2).

## Discussion

The present study reported the recent twelve-year epidemiological profile of circulating HAdV strains in children with ARI at a regional children’s medical service network hospital in Guangzhou, China. The infection rate varies in different years. A previous study of 1778 hospitalized children with pneumonia in Guangzhou showed an infection rate of 2.50% from 2013 to 2017 and 6.00% from 2018 to 2019 [16]. Our study showed that the annual HAdV infection frequency in the Guangzhou area ranged from 3.92% (2012) to 13.58% (2019) between 2010 and 2019 before the COVID-19 pandemic. Our data were collected during a twelve-year research period, where at least 4500 samples were tested annually with more than 200 samples tested almost every month. This sampling period and study duration can accurately reflect the incidence of adenovirus infections in children. From 2010 to 2019, two years (2014 and 2019) were big epidemic years, suggesting that HAdV epidemics might peak every four or five years. These results provide a reference for predicting the time of the next epidemic peak in the Guangzhou area. At the same time, our data also showed that the HAdV epidemic in the Guangzhou area has a distinct seasonal distribution, with a prominent activity peak in July and August every year and the lowest activity around March.

We did not observe any gender difference in HAdV infection, which is consistent with previous studies conducted in other provinces and countries [16, 17]. The highest HAdV positive proportions were observed among children over six years of age, which was similar to some other studies done in Guangzhou [16, 18]. A study on seroprevalence and titer levels of neutralizing antibodies (NAb) against HAdV in healthy populations in 2017 from Guangzhou showed that the HAdV antibodies in children over five years were higher than those in other age groups, which was consistent with the results of the present study [19].

We have reported previously that HAdV-3 was responsible for most of the HAdV infection among hospitalized children in Guangzhou between 2012 and 2013 [12]. As an extension of that study, we found that HAdV-3 was still the main subtype until early 2017. The proportion of HAdV-7 infections began to rise in the second half of 2017, and by 2018 and 2019 HAdV-7 infections among hospitalized children had risen to more than 80%.

The large epidemic in 2019 led to a sharp increase in pediatric patients in the region. In that year nearly 40,000 children were tested for HAdV in our laboratory, with a positive rate of 13.58%, which was the highest in the past decade. This epidemic caused a shortage of hospital beds, and many children could not be hospitalized. Thus, 37.86% of adenovirus-positive cases detected in 2019 came from outpatients. From 2018 to 2019, not only the Guangzhou area but also Hubei, Wenzhou and other places in Southern and Eastern China also witnessed a HAdV epidemic among children [20, 21] while Northern China did not. In Northern China HAdV-3 was the most common subtype followed by HAdV-7 from 2018 to 2019 [22, 23]. According to our research, from 2012 to 2016, HAdV-3 was the dominant subtype in Guangzhou, and HAdV-7 accounted for a relatively small proportion. Consequently, HAdV-7 immunity in children was likely low. A study in Guangzhou showed that the seroprevalence of neutralizing antibodies against HAdV-7 (10.90%) was much lower than HAdV-3 (29.40%) under the age of 18 in 2017 [19]. Some research revealed that HAdV subtype antisera showed no neutralizing activity to other subtype [4]. Immunity against HAdV-3 probably does not protect against HAdV-7. It is possible that the circulation of different subtypes may help these viruses to escape the pre-existing immune response.

HAdV-7 was detected in a higher proportion of HAdV infections in the intensive care units, even when HAdV-7 was not the prevalent strain in circulation that year. This is also consistent with reports that HAdV-7 infection is usually more severe than that of HAdV-3 [11, 24, 25]. Our data indicate the same, as 93% of the 29 fatal cases who were adenovirus positive in the ICU were HAdV-7. If infected with adenoviruses, the mortality proportion in children ranges from 0.28% to 0.47% but rises to about 20% in severe cases [26]. During the adenovirus epidemic from 2018 to 2019, there was a sharp increase in the number of children with severe cases in 2018, and the mortality proportion was relatively high (0.47%). However, from the experiences gained in treating severely infected children in 2018, our hospital began to use extracorporeal membrane oxygenation (ECMO) to rescue severe patients, which resulted in a slightly improved mortality proportion of severe adenovirus patients (0.28%) in 2019.

Genome variation analysis displayed the stable genome of HAdV-3 and HAdV-7 between 2018 and 2019 [20]. Sequence variation analysis indicated that three genes (*penton base*, *hexon*, and *fiber*) of HAdV-7 were relatively stable across time and geographic space, particularly for viruses within sub-types, which shared almost the same variation sites [27]. Molecular typing of human adenoviruses among hospitalized patients with RTI (respiratory tract infection) in Guangzhou between 2017 and 2019 indicates stable conservation of HAdV-3 and -7 [18]. Other research showed the nucleotide sequence homologies of the *hexon*, *fiber* and *penton* gene for HAdV-7 and HAdV-3 in Guangzhou between 2013 and 2019 were high (99.5–100%) [16], suggesting low strain variation among the circulating viruses. HAdV-7d should be of particular concern within the HAdV subtype. HAdV-7d was believed to be closely related to this HAdV epidemic in Southern China. In Hubei, a province in Southern China, restriction endonuclease analysis (REA) revealed that HAdV-7 belong to genome 7d [20]. We also detected HAdV-7d in the fatal cases, which had been reported to cause severe illness [5]. Between 2016 and 2019, we also detected HAdV-55 which caused a few severe cases. This study suggests that HAdV-55 has become a common pathogen causing life-threatening pneumonia in Guangzhou after the first outbreak in China in 2006 [28].

Human adenoviruses are classified into seven species (A–G) and at least 110 genotypes, as defined by the Human Adenovirus Working Group (http://hadvwg.gmu.edu/). HAdV direct or indirect transmission can occur through the throat, feces, eyes or urine, depending on the virus subtype. Certain HAdV subtypes are predominantly associated with specific pathologies, such as acute respiratory outbreaks (HAdV-B/C/E) [8, 21], epidemic keratoconjunctivitis (HAdV-D) [29, 30], gastroenteritis, and/or acute hemorrhagic cystitis (HAdV-F/G) [31, 32]. On 31 March 2022 Public Health Scotland reported a cluster of cases of severe hepatitis of unknown origin in Scotland. Five children were adenovirus PCR-positive, which suggested that adenovirus also may be a possible pathogen causing unexplained severe acute liver injury in children [33].

The present study has some limitations. First, due to the nature of the retrospective analysis, some data were incomplete or missing. For example, the demographic data of HAdV infection before 2014 and the number of ICU cases before 2015 were not available because they were not collected. Second, HAdV sequencing was not carried out in the early samples. With the increasing understanding of adenovirus infection in children, we detected HAdV subtypes in the respiratory ward in 2012, and in the ICU ward in 2014. Third, considering the major HAdV subtypes in China and the relatively limited resources, we only detected the main common HAdV subtypes and classified the remaining unknown samples as other HAdV subtypes. Therefore, there is no in-depth discussion on other HAdV subtypes in this study. Finally, it is noteworthy that HAdV was effectively reduced after the emergence of SARS-CoV-2 infection in 2020. China implemented stringent non-pharmaceutical interventions (NPIs) in spring 2020 as part of the response to the emergence of SARS-CoV-2. In our study HAdV activities were reduced significantly during this period. This suggested that NPIs adopted to manage COVID-19 were also effective in preventing transmission of HAdV.

In conclusion, our study highlights the transmission pattern and epidemiology of HAdV-infections in children in the Guangzhou area within the last 12 years. These data indicate that there was a shift in the dominant HAdV strain from HAdV-3 to HAdV-7 in 2018 and 2019 in our hospital, which may have been reflected in Guangzhou as a whole. HAdV-7 is associated with more severe disease and, as our data indicated that HAdV-3 was the dominant subtype, there may have also been limited immunity against HAdV-7, which could have been a contributing factor to the outbreak. Our findings suggest that the transmission pattern of HAdV-types may be associated with population immunity and should be investigated in future studies. Continued surveillance will be essential to predict the epidemic sizes, plan for adequate management of health care resources and to develop vaccines to combat future outbreaks.

## Data Availability

Condensed anonymized data are available from the corresponding author on reasonable request.
